# Subtraction Maps Derived from Longitudinal Magnetic Resonance Imaging in Patients with Glioma Facilitate Early Detection of Tumor Progression

**DOI:** 10.3390/cancers12113111

**Published:** 2020-10-24

**Authors:** Nico Sollmann, Magaly Gutbrod-Fernandez, Egon Burian, Isabelle Riederer, Bernhard Meyer, Andreas Hock, Jens Gempt, Claus Zimmer, Jan S. Kirschke

**Affiliations:** 1 Department of Diagnostic and Interventional Neuroradiology, Klinikum rechts der Isar, Technische Universität München, Ismaninger Str. 22, 81675 Munich, Germany; Fernandez.S.Magaly@googlemail.com (M.G.-F.); Egon.Burian@tum.de (E.B.); Isabelle.Riederer@tum.de (I.R.); Claus.Zimmer@tum.de (C.Z.); Jan.Kirschke@tum.de (J.S.K.); 2 TUM-Neuroimaging Center, Klinikum rechts der Isar, Technische Universität München, 81675 Munich, Germany; 3 Department of Neurosurgery, Klinikum rechts der Isar, Technische Universität München, Ismaninger Str. 22, 81675 Munich, Germany; Bernhard.Meyer@tum.de (B.M.); Jens.Gempt@tum.de (J.G.); 4 Health Systems Philips Switzerland, Seestrasse 87, 8810 Horgen, Switzerland; Andreas.Hock@philips.com; 5 TranslaTUM—Central Institute for Translational Cancer Research, Klinikum rechts der Isar, Technische Universität München, 81675 Munich, Germany

**Keywords:** brain neoplasms, disease progression, glioma, magnetic resonance imaging, subtraction technique

## Abstract

**Simple Summary:**

Tumor recurrence is common among World Health Organization (WHO) grade II to IV gliomas. Magnetic resonance imaging (MRI) including fluid attenuated inversion recovery (FLAIR) sequences is key to detection of recurrence. This study used subtraction maps (SMs) derived from serial FLAIR imaging in 100 patients with glioma to facilitate detection of progressive disease, compared to conventional (CONV) visual reading. Reading of SMs revealed FLAIR signal increases in a larger proportion of patients and with higher diagnostic confidence according to evaluations of two readers. Correspondingly, an improved sensitivity (99.9% vs. 73.3%) was observed for SM reading when compared to CONV reading.

**Abstract:**

Progression of glioma is frequently characterized by increases or enhanced spread of a hyperintensity in fluid attenuated inversion recovery (FLAIR) sequences. However, changes in FLAIR signal over time can be subtle, and conventional (CONV) visual reading is time-consuming. The purpose of this monocentric, retrospective study was to compare CONV reading to reading of subtraction maps (SMs) for serial FLAIR imaging. FLAIR datasets of cranial 3-Tesla magnetic resonance imaging (MRI), acquired at two different time points (mean inter-scan interval: 5.4 ± 1.9 months), were considered per patient in a consecutive series of 100 patients (mean age: 49.0 ± 13.7 years) diagnosed with glioma (19 glioma World Health Organization [WHO] grade I and II, 81 glioma WHO grade III and IV). Two readers (R1 and R2) performed CONV and SM reading by assessing overall image quality and artifacts, alterations in tumor-associated FLAIR signal over time (stable/unchanged or progressive) including diagnostic confidence (1—very high to 5—very low diagnostic confidence), and time needed for reading. Gold-standard (GS) reading, including all available clinical and imaging information, was performed by a senior reader, revealing progressive FLAIR signal in 61 patients (tumor progression or recurrence in 38 patients, pseudoprogression in 10 patients, and unclear in the remaining 13 patients). SM reading used an officially certified and commercially available algorithm performing semi-automatic coregistration, intensity normalization, and color-coding to generate individual SMs. The approach of SM reading revealed FLAIR signal increases in a larger proportion of patients according to evaluations of both readers (R1: 61 patients/R2: 60 patients identified with FLAIR signal increase vs. R1: 45 patients/R2: 44 patients for CONV reading) with significantly higher diagnostic confidence (R1: 1.29 ± 0.48, R2: 1.26 ± 0.44 vs. R1: 1.73 ± 0.80, R2: 1.82 ± 0.85; *p* < 0.0001). This resulted in increased sensitivity (99.9% vs. 73.3%) with maintained high specificity (98.1% vs. 98.8%) for SM reading when compared to CONV reading. Furthermore, the time needed for SM reading was significantly lower compared to CONV assessments (*p* < 0.0001). In conclusion, SM reading may improve diagnostic accuracy and sensitivity while reducing reading time, thus potentially enabling earlier detection of disease progression.

## 1. Introduction

Gliomas arise in the glial tissue and represent the most common malignant brain tumor type in adults, with an average annual age-adjusted incidence rate of approximately 4.67 to 5.73 per 100,000 population [1,2]. Depending on the molecular and histology profiles, gliomas can be categorized according to the World Health Organization (WHO) grades [3,4]. Anaplastic astrocytoma (WHO grade III) and glioblastoma (WHO grade IV) are considered the major representatives of high-grade glioma and have shown increases in incidence with age, peaking in elderly subjects [1,2,5]. Oligodendroglioma (WHO grade II) and diffuse astrocytoma (WHO grade II) are common low-grade glioma entities that typically occur earlier in adulthood [1,2,5]. WHO grade II to IV gliomas are considered chronic progressive diseases related to their diffuse and infiltrative growth patterns, making curative treatment mostly impossible [5,6].

Associated with the infiltrative growth pattern, tumor recurrence is common among WHO grade II to IV gliomas and can also infrequently occur in WHO grade I gliomas. Even in cases initially showing complete macroscopic removal after neurosurgical resection as the first-line treatment, early and late progression can be observed particularly in WHO grade II to IV gliomas [6,7,8,9]. Early detection of tumor recurrence is crucial to initiate further treatment, with repeated resection often being a feasible option to warrant optimal outcome in terms of survival [10,11,12,13,14]. Resections can be complemented by adjuvant radiotherapy (RTX) and/or chemotherapy (CTX), or such treatment can be considered a stand-alone option for tumors that are not operated on (e.g., due to eloquent tumor location) [15,16,17,18]. Regarding pre- and post-treatment diagnostics, magnetic resonance imaging (MRI) represents the gold-standard (GS) technique to detect glioma and disease progression [19,20].

Due to considerable individual variance in the time point of recurrence, if any, multiple follow-up (FU) imaging studies are scheduled in clinical routine. Fluid attenuated inversion recovery (FLAIR) sequences are integral for FU imaging in both low- and high-grade glioma as disease progression manifests in increases of FLAIR hyperintensity, alongside with typically increasing or reoccurring contrast enhancement in high-grade glioma or malignant transformation [19,21,22,23]. Particularly in low-grade glioma that commonly do not show contrast enhancement on T1-weighted imaging, FLAIR sequences are key to determine the state of disease and to detect early progression [19,21,22,23]. However, changes in the FLAIR signal over time can be subtle, and conventional (CONV) reading by visually comparing longitudinal FLAIR imaging can be very time-consuming.

Subtraction maps (SMs) for evaluation of serial MRI examinations have previously shown to reduce reading time and to improve accuracy for FU imaging in multiple sclerosis [24,25,26,27,28]. However, in neurooncology, approaches using SMs especially for detection of early, thus subtle disease progression that can be seamlessly used in clinical routine are mostly lacking to date. The aim of this study was to systematically investigate a commercially available software application for SM generation and reading in routine glioma imaging.

## 2. Materials and Methods

### 2.1. Study Design and Patient Inclusion

This retrospective, monocentric study was approved by the local institutional review board (Technical University of Munich, registration number: 340/16 S) and was in accordance with the Declaration of Helsinki. Written informed consent was not required for this study because of its retrospective character.

We retrospectively identified eligible patients via searching our institutional Picture Archiving and Communication System (PACS) considering the following inclusion criteria: (1) age above 18 years, (2) availability of at least two MRI acquisitions from two different FU time points (defined as MRI_1 and MRI_2) performed at our institution using our standard glioma imaging protocol (including FLAIR imaging), (3) inter-scan interval of at least four weeks between MRI_1 and MRI_2, (4) diagnosis of glioma (WHO grades I–IV) according to histopathological evaluation (based on previous surgery or biopsy), and (5) supratentorial location of the tumor mass. The following criteria led to exclusion of patients: (1) tumor resection or biopsy performed within the inter-scan interval between MRI_1 and MRI_2, (2) non-diagnostic image quality of MRI datasets according to visual assessment, (3) artifacts due to foreign bodies or motion artifacts in imaging data, (4) other coexisting intracranial structural pathologies (except for glioma), and (5) pregnancy.

The PACS search considered patients who presented at our institution for FU imaging between May 2019 and January 2020, with the last MRI scan acquired in this period representing MRI_2. Imaging data defined as MRI_1 were acquired between November 2018 and September 2019. All MRI scanning was performed according to medical indication for tumor FU imaging during clinical routine. Patient characteristics and medical history was extracted from electronic health records. Overall, 200 MRI datasets derived from a consecutive series of 100 patients fulfilling the inclusion criteria were used in this study.

### 2.2. Magnetic Resonance Imaging

Cranial MRI was acquired in supine position on a 3-Tesla scanner (Achieva dStream or Ingenia; Philips Healthcare, Best, The Netherlands) using a 32-channel head coil. Both scanning for MRI_1 and MRI_2 was performed with the identical imaging protocol. The standardized protocol for brain tumors at our institution includes, amongst further dedicated sequences, a three-dimensional (3D) FLAIR sequence that covers the whole head with the following parameters: repetition time/echo time = 4800/274 ms, inversion time = 1650 ms, echo train length = 170, number of phase encoding steps = 250, flip angle = 90°, acquisition matrix = 0/250/250/0, voxel size = 1 × 1 × 1 mm^3^, parallel imaging using Compressed SENSE, acquisition duration ~4 min.

### 2.3. Conventional and Subtraction Map Reading

#### 2.3.1. Setup and Scoring

Two readers (R1 and R2, with eight and three years of experience in neuroradiology) performed CONV and SM reading using the PACS viewer (IDS7; Sectra AB, Linköping, Sweden). As preparation for evaluation, image layouts and SMs were generated by a third person not involved in image reading. Generation of the individual SM took less than five minutes on average per patient.

Evaluations were performed according to a standardized scoring scheme (Table 1), which covered overall image quality (for CONV reading), overall artifacts (for SM reading), and diagnostic confidence (separately for CONV reading and SM reading). Evaluation of overall artifacts during SM reading was included to capture any image errors introduced by the SM generation procedure (e.g., incorrect coregistration of sequences or issues of color scaling with widespread erroneous coloring of brain tissue not related to tumor progression or pseudoprogression). Furthermore, the FLAIR signal was determined as either progressive or stable/unchanged using binary grading (0–stable/unchanged, 1–progressive). Additionally, the time needed per patient and reading was recorded.

Patient cases were assessed in randomized order after pseudonymization, and readers were blinded to the results of evaluation of each other as well as to radiological reports created during clinical routine and the electronic health records of the patients. Furthermore, they were strictly blinded to any previous or later imaging in relation to MRI_1 and MRI_2 as well as to other sequences than FLAIR imaging.

#### 2.3.2. Conventional Reading

**Figure 1:**
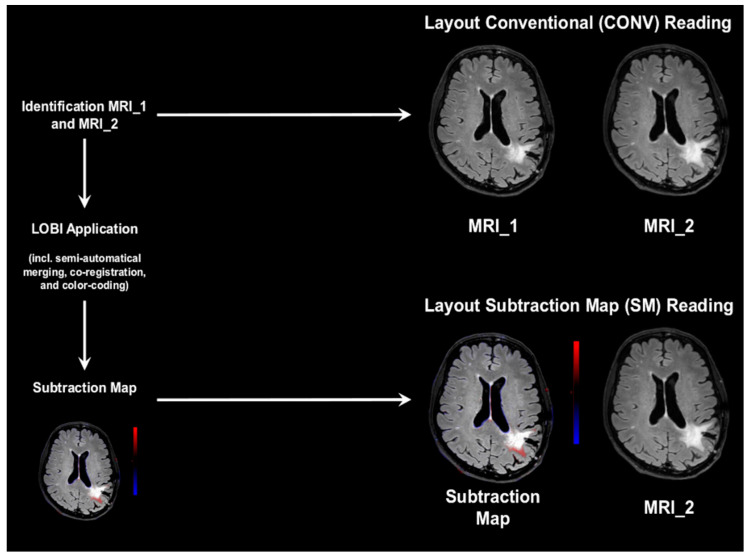
Schematic illustration of image preparation and layouts. Image layouts were generated for conventional (CONV) reading and subtraction map (SM) reading, respectively. For CONV reading, the layout depicted the fluid attenuated inversion recovery (FLAIR) sequences of MRI_1 and MRI_2 alongside each other, and the readers performed standard visual reading of the image pairs. For SM reading, a color-coded SM was created first based on the FLAIR sequences of MRI_1 and MRI_2. The layout for SM reading included the individual SM alongside with MRI_2. In the SMs, new or progressive lesions were shown in red, and regressive lesions were depicted in blue.

For CONV assessment, a layout showing axial slices of the FLAIR sequences of MRI_1 and MRI_2 alongside each other was used (Figure 1). Readers performed visual reading of the image pairs without any computer assistance, as it is commonly performed during clinical routine. Readers were allowed to manually reformat and reangulate images and to consider axial, coronal, and sagittal planes (with 1 mm slice thickness).

#### 2.3.3. Subtraction Map Reading

After an interval of at least four weeks, SM reading was performed. To generate SMs, a commercially available application implemented in IntelliSpace Portal was used (Longitudinal Brain Imaging [LOBI]; Philips Healthcare, Best, The Netherlands), which has obtained certification in line with the standards of the Conformité Européenne (CE) and United States Food and Drug Administration (FDA). After unprocessed imaging data of the FLAIR sequences for MRI_1 and MRI_2 were selected and sent to a local server, the application provided an automated approach consisting of multiple steps for image processing. In detail, it performs bias field correction, followed by merging and rigid coregistration of both sequences using an affine registration approach (Figure 1). Additionally, intensity scaling is implemented to adjust for potential differences in signal intensities between sequences. Processing continues with a subtraction of the images, followed by generation of overlays of the subtraction results as a color coding to the baseline image to create the final SM (Figure 1).

Progressive or new lesions are shown as red in such maps, while regressive lesions are depicted in blue by default. The SMs were transferred back to PACS from the local server, and a layout with this map side-by-side with axial slices of the FLAIR sequence of MRI_2 was created per patient. Again, readers were allowed to consider all three planes (with 1 mm slice thickness) during image evaluations.

### 2.4. Gold-Standard Reading

GS reading was independently performed by a senior consultant (board-certified radiologist with eight years of experience in neuroradiology). The GS reading was performed for two purposes: (1) to discriminate between FLAIR signal alterations due to tumor progression and changes related to pseudoprogression (i.e., in the context of treatment-associated effects), if possible, and (2) to determine longitudinal FLAIR signal progression or unchanged FLAIR signal, particularly by incorporating long-term FU imaging beyond MRI_1 and MRI_2. In cases with very subtle FLAIR signal changes between MRI_1 and MRI_2, changes were considered to be stable when remaining stable in subsequent FU examinations, and considered to be progressive if they were clearly enlarged in subsequent FU images or progressive disease was diagnosed by positron emission tomography (PET).

Besides FLAIR sequences of MRI_1 and MRI_2 and related SMs, GS reading included all other imaging sequences available for these acquisitions and all other imaging data available in PACS per patient (non-contrast- and contrast-enhanced MRI and PET, if available), as well as the entire medical history including information on adjuvant CTX and/or RTX. Furthermore, histopathological evaluation in case of re-resection or re-biopsy of suspected tumor recurrence was considered, as well as the discussions and decisions of an interdisciplinary tumor board (including neuroradiologists, neurosurgeons, neurologists, and radiation oncologists).

### 2.5. Statistical Analysis

For statistical analyses, GraphPad Prism (version 6.0; GraphPad Software Inc., San Diego, CA, USA) and SPSS (version 20.0; IBM SPSS Statistics for Windows, IBM Corp., Armonk, NY, USA) were used. A *p*-value < 0.05 was considered statistically significant.

Descriptive statistics were computed for patient-related characteristics and scorings. Shapiro-Wilk normality tests indicated non-Gaussian distribution for the majority of data. Chi-Squared tests were applied to assess differences in distributions of detected progressive or stable FLAIR signal alterations. Wilcoxon matched-pairs signed rank tests were performed on reading times and diagnostic confidence to compare CONV against SM reading. To assess intra- or inter-reader agreement, weighted Cohen’s kappa (*κ*) was calculated for scorings of R1 and R2.

Furthermore, receiver operating characteristics (ROC) were calculated for the whole cohort of patients using the following definitions based on progressive or stable FLAIR signal alterations: true positive = progressive FLAIR signal in GS reading AND CONV or SM reading, true negative = stable FLAIR signal in GS reading AND CONV or SM reading, false positive = stable FLAIR signal in GS reading AND progressive FLAIR signal in CONV or SM reading, false negative = progressive FLAIR signal in GS reading AND stable FLAIR signal in CONV or SM reading. In addition, sensitivity was also calculated for patients who showed tumor progression, thus excluding patients with pseudoprogression or unclear entity of FLAIR signal alterations over time.

## 3. Results

### 3.1. Patient Cohort

This study included 100 patients (mean age: 49.0 ± 13.7 years, age range: 18.8–79.9 years, 55 males) diagnosed with supratentorial glioma (WHO grades I–IV), who contributed 100 pairs (MRI_1 and MRI_2) of FLAIR imaging (Table 2).

### 3.2. Image Quality and Artifacts

On average, image quality was rated as good to perfect by both readers (R1: 1.55 ± 0.59, R2: 1.52 ± 0.65, inter-reader *κ* = 0.83). Only minimal artifacts were observed in individual SMs according to both readers (R1: 1.67 ± 0.71, R2: 1.68 ± 0.72, inter-reader *κ* = 0.96).

### 3.3. Evaluation of FLAIR Signal Changes

#### 3.3.1. Progressive Versus Stable FLAIR Signal

**Figure 2:**
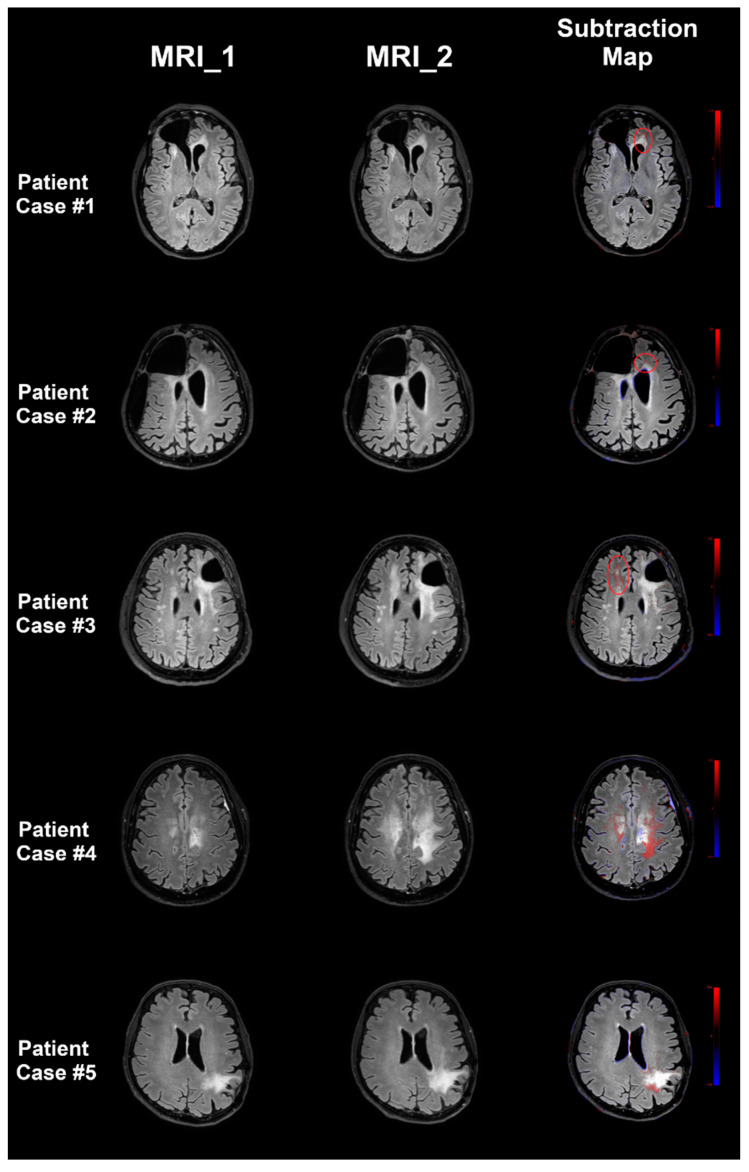
Exemplary patient cases. Five exemplary patient cases with increasing fluid attenuated inversion recovery (FLAIR) signal, thus suspected (pseudo)progression, are shown in this figure. Axial slices of the FLAIR sequences of MRI_1 and MRI_2 as well as the subtraction maps (SMs) are shown, which use color-coding to show signal changes over time (new or progressive lesions are shown in red). For patient cases #1, #2, and #3, progressive FLAIR signal is rather subtle and is enclosed by red circles (located adjacent to the left-hemispheric anterior horn of the lateral ventricle for patient case #1, in the left-hemispheric frontal white matter close to an enlarged left-sided lateral ventricle in patient case #2, and in the right-hemispheric frontal white matter close to the lateral ventricle in patient case #3). In patient cases #4 and #5, progressive FLAIR signal was more extensive, and thus also easier to detect without SMs when comparing MRI_1 and MRI_2 (located in the bihemispheric centrum semiovale for patient case #4 and in the left-hemispheric parietal white matter extending to the lateral ventricle in patient case #5). For patient cases #1, #2, and #3, the FLAIR signal was determined as stable/unchanged by one reader each during conventional (CONV) reading that only used MRI_1 and MRI_2, whereas it was categorized as progressive by both readers according to the SM reading. The FLAIR signal for patient cases #4 and #5 was rated as progressive by both readers during CONV and SM reading, respectively.

**Figure 3:**
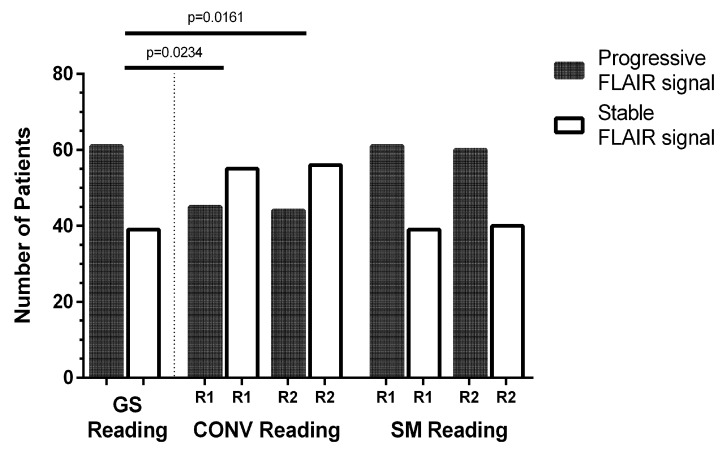
Evaluation of signal changes—stable versus progressive signal. Gold-standard (GS) reading defined 61 patients to show increases of the fluid attenuated inversion recovery (FLAIR) signal and the remaining 39 patients to show stable FLAIR signal alterations. Subtraction map (SM) reading was in good accordance with GS reading according to both readers (R1 and R2), with 61 patients (R1)/60 patients (R2) being correctly identified to show progressive FLAIR signal (R1: *p* = 1.0000, R2: *p* = 0.8850). Increasing FLAIR signal for 16 patients (R1)/17 patients (R2) was missed in conventional (CONV) reading (R1: *p* = 0.0234, R2: *p* = 0.0161).

Figure 2 depicts five exemplary patient cases with increasing FLAIR signal. According to GS reading, 61 patients showed progression of the FLAIR signal, while 39 patients showed stable FLAIR signal alterations (Figure 3). SM reading was close to the GS, with 61 patients (R1)/60 patients (R2) being correctly identified to show progressive FLAIR signal (R1: *p* = 1.0000, R2: *p* = 0.8850). Increasing FLAIR signal for 16 patients (R1)/17 patients (R2) was missed in CONV reading (R1: *p* = 0.0234, R2: *p* = 0.0161). Inter-reader agreement was high for CONV reading (*κ* = 0.94) and SM reading (*κ* = 0.98).

#### 3.3.2. Tumor Progression Versus Pseudoprogression

**Figure 4:**
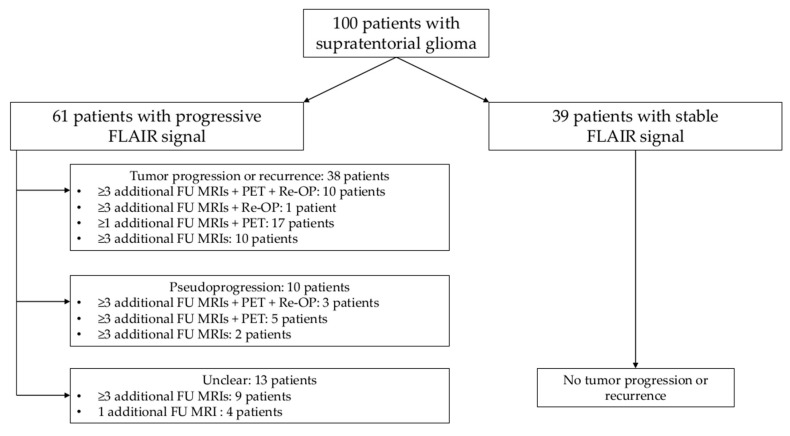
Evaluation of signal changes—progression versus pseudoprogression. This chart shows the number of patients with progressive or stable fluid attenuated inversion recovery (FLAIR) signal. Furthermore, for the patients with increasing signal alterations, the differentiation between tumor progression, pseudoprogression, and unclear cases is depicted, which was based on gold-standard (GS) reading using FLAIR sequences of MRI_1 and MRI_2, all other imaging sequences of these and any other imaging sessions (non-contrast- and contrast-enhanced MRI and positron emission tomography [PET], if available), as well as the entire medical history including information on adjuvant chemotherapy (CTX) and/or radiotherapy (RTX). Histopathological evaluation (in case of re-resection or re-biopsy of suspected tumor recurrence) and the decisions of an interdisciplinary tumor board were considered as well.

Among the 61 patients who showed progression of the FLAIR signal, GS reading indicated tumor progression or recurrence in 38 patients and pseudoprogression in 10 patients, while increasing FLAIR signal alterations of 13 patients remained unclear (Figure 4). All assessed MRI scans included contrast-enhanced T1-weighted imaging as well as dynamic susceptibility contrast perfusion imaging.

#### 3.3.3. ROC Analysis

Average sensitivity was higher for SM reading when compared to CONV reading (99.9% versus 73.3%), whereas there was no clear difference for specificity (98.1% versus 98.8%; Table 3). When only investigating patients with tumor progression (thus excluding patients with pseudoprogression or unclear entity of FLAIR signal alterations), sensitivity remained largely unchanged for SM reading compared to CONV reading (99.9% versus 73.7%).

### 3.4. Reading Time and Diagnostic Confidence

The time needed for SM reading was significantly lower when compared to CONV assessment according to both R1 (CONV: 2.40 ± 0.77 min, SM: 0.95 ± 0.36, *p* < 0.0001) and R2 (CONV: 2.20 ± 0.69 min, SM: 0.84 ± 0.40, *p* < 0.0001). At the same time, diagnostic confidence was rated significantly better (*p* < 0.0001) for SM reading (R1: 1.29 ± 0.48, R2: 1.26 ± 0.44) compared to CONV reading (R1: 1.73 ± 0.80, R2: 1.82 ± 0.85).

## 4. Discussion

This study investigated a commercially available, CE-certified and FDA-approved software application for SM generation and reading for longitudinal glioma imaging based on FLAIR image pairs. The main findings were higher accuracy, sensitivity, and diagnostic confidence for SM reading, alongside with significantly reduced time required for image assessments when compared to CONV reading.

For radiologists, providing diagnostics with the highest level of accuracy in days of ever rising exam numbers becomes more and more challenging. Steadily decreasing scan times and higher spatial resolution due to technical advances, such as parallel imaging and higher field strengths introduced into clinical routine, further push boundaries for daily caseloads [29,30,31]. Despite an evident need for compensation strategies, recent high innovation on the MRI technology side and related demands remain mostly unanswered by diagnostic image assessment, which is still widely based on simple visual reading. In keeping with this imbalance, we are aware of only one previous study for neurooncological imaging that evaluated an approach based on SMs that was seamlessly integrated in the routine setting [32]. However, in this previous retrospective study, a smaller cohort consisting of 41 patients with different entities of glioma was considered, and these patients were scanned with various, inhomogeneous MRI protocols on various scanners from different manufacturers, with field strengths of scanners ranging between 1 and 3 Tesla [32]. As acknowledged by the authors of this previous work, considering examinations from different MRI scanners and with different sequence parameters and slice thicknesses may result in partial volume effects that may hamper image post-processing methods [32]. Moreover, such differences in image acquisition protocols may considerably bias correct identification of early tumor progression, pointing to the need for more robust data. Against this background, the present study provides data taken exclusively from 3-Tesla MRI systems of the same vendor using an identical sequence with high spatial resolution across all patients.

Furthermore, two studies applied the same algorithm as used in this study to longitudinal imaging in patients with multiple sclerosis, demonstrating significant reductions in reading time as well as improved diagnostic accuracy for lesion detection [24,25]. Other studies using comparable, not necessarily officially approved applications also reported on higher detection rates for new lesions in multiple sclerosis when set in relation to standard reading approaches [26,27,28]. Furthermore, in patients who underwent cardiac surgery, a fully automated algorithm deploying coregistration and bias field correction enabled detection of more ischemic lesions with reduced reading time compared to standard assessments [33]. Similarly, the present study revealed increased diagnostic accuracy and sensitivity for diagnostics based on SMs, although in a cohort of patients with glioma.

Major advantages of the SM reading over CONV reading are semi-automatic coregistration and color coding, likely both contributing to increased accuracy and sensitivity. Regarding the almost perfect ROC metrics for SM reading, we have to acknowledge that dedicated neuroradiologists used to high neurooncological caseloads at a specialized academic center performed evaluations for MRI_1 and MRI_2 with identical sequence parameters, thus exploiting maximum benefit from the SM implementation. While this approach may serve as proof of concept, longitudinal imaging in a clinical routine is frequently confronted with MRI datasets from different scanners and acquisitions with various protocols, which could negatively interfere with the SM generation algorithm. On the other hand, average inter-scan intervals between MRI_1 and MRI_2 were rather short in this study, thus probably more likely exposing readers to subtle changes that are at special risk to be missed. Considering these circumstances, SM reading was nevertheless able to outperform CONV reading.

Regarding longitudinal imaging of patients diagnosed with glioma, assessment of FLAIR signal alterations over time is of particular interest given that progression of tumor tissue frequently occurs earlier than clinical progression [34,35]. Specifically, changes in non-enhancing tumor burden can be identified in FLAIR imaging even when progression is in an early stage and, thus, only becomes manifest in subtle signal changes. However, it has to be emphasized that FLAIR imaging should be considered one integral part of a multi-sequence protocol for imaging in neurooncological patients, with other sequences also heavily contributing to correct diagnosis in initial as well as FU exams of glioma [19]. Furthermore, the clinical value of single or combinations of multiple sequences may be facilitated by correlating imaging findings to prognosis. With regard to the approach presented in this study, prospective FU studies may evaluate the progression in FLAIR signal alterations, detected by SMs but not during CONV reading, to predict prognosis. This kind of evaluation may potentially allow to determine the minimum volume of FLAIR signal alteration a radiologist can correctly detect during visual reading, thus further exploiting SM reading and utility in clinical practice.

When interpreting the results of this study we have to acknowledge the following limitations. First, we performed straightforward binary categorization for FLAIR signal alterations, thus not considering tumor volumetry. In this context, it has been shown previously by a semi-automatic segmentation approach for disease progression that changes in FLAIR volume have better accuracy than regional, segmentation-based subtractions [36]. Implementation of such (semi-)automated procedures for tumor volumetry have, however, not yet been made available for broad use during clinical routine. Second, while the time needed for SM reading was significantly lower when compared to CONV reading, one has to be aware of the requirement of dedicated SM generation (less than five minutes on average per patient in our study), which also takes time and user interaction. However, automated forwarding of image sequences directly from the MRI scanner or from PACS to the application for creation of SMs seems feasible with limited efforts, which should ideally be supplemented by fully automatic initiation of SM generation. Yet, this pipeline is not routinely established in the workflow of this application. Third, we did not exclusively enroll patients with confirmed tumor progression or pseudoprogession, which is a common and inherently heterogeneous imaging-based differential diagnosis of actual tumor growth [37,38]. While the origin of increasing FLAIR signal alterations was not relevant to the binary scoring for CONV and SM reading, we have to be aware that even in the light of GS reading, not all cases of this study were classifiable as progressive diseases or pseudoprogession with last certainty. Yet, novel approaches using machine learning or radiomic features for processing and interpretation of MRI data in patients harboring glioma may improve differentiation of treatment-related effects from actual tumor recurrence [39,40,41]. However, while such approaches are promising and may considerably support correct interpretation in the future, visual reading still resembles daily clinical situations where unequivocal diagnosis, particularly in patients with limited series of FU imaging, is not always possible. A combination of machine learning or radiomic features with SM evaluations may have potential for upcoming trials in neurooncological imaging, where the yield of the SM approach may be even further increased.

## 5. Conclusions

Using SM reading in longitudinal imaging of glioma may considerably improve diagnostic accuracy and sensitivity with reduced time needed for image evaluation. This result is obtained with a CE-certified and FDA-approved software application, thus pointing at ultimate benefit for neurooncological diagnostics in clinical routine.

## Figures and Tables

**Table 1:** Scoring system for visual image reading.

Qualitative Image Evaluation					
Item	Score				
	1	2	3	4	5
**Overall Image Quality** (for CONV reading)	Very good to perfect quality	Good to very good quality	Medium quality	Adequate quality	Poor quality
**Overall Artifacts** (for SM reading)	No artifacts	Minimal artifacts	Prominent artifacts	Major artifacts	Severe artifacts
**Diagnostic Confidence** (for CONV and SM reading)	Very high	High	Intermediate	Low	Very low

This table provides an overview of the scoring system, covering overall image quality, overall artifacts, and diagnostic confidence. Rating of overall image quality was performed for conventional (CONV) reading (evaluating the fluid attenuated inversion recovery [FLAIR] sequences of MRI_1 and MRI_2), while scoring for overall artifacts was done for the subtraction map (SM) reading (thus evaluating the color coding as overlaid on the FLAIR sequence of MRI_1). In addition to this scoring system, the FLAIR signal was determined as either progressive or stable/unchanged (0–stable/unchanged, 1–progressive), and the time needed per patient and reading was recorded.

**Table 2:** Cohort characteristics.

Cohort Characteristics			
**Tumor entity** (N, patients)	Glioma WHO grade I		2
	Glioma WHO grade II		17
	Glioma WHO grade III		42
	Glioma WHO grade IV		39
**Tumor location** (N, patients)	Left hemisphere		59
	Right hemisphere		37
	Multifocal/Corpus callosum		4
**Time since first diagnosis** (months, mean ± SD [range])			45.8 ± 58.7 [1.1–334.1]
**Tumor resection/biopsy performed** (N, patients)			96/4
**Time since (last) tumor resection** (months, mean ± SD [range])			20.9 ± 22.1 [1.0–114.8]
**Interval between MRI_1 and MRI_2** (months, mean ± SD [range])			5.4 ± 1.9 [1.0–9.6]
**Adjuvant** **CTX**	**CTX performed** (N, patients)		89
	**Substance of (last) CTX** (N, patients)	Temozolomide	64
		PCV	14
		CCNU	10
		Bevacizumab	1
	**Time since (last) CTX** (months, mean ± SD [range])		14.4 ± 16.8 [0.3–72.2]
**Adjuvant** **RTX**	**RTX performed** (N, patients)		93
	**RTX dose of (last) RTX** (Gy, mean ± SD [range])		53.5 ± 8.8 [20.0–64.0]
	**Time since (last) RTX** (months, mean ± SD [range])		27.7 ± 44.7 [0.1–334.1]

This table provides an overview of cohort characteristics including demographics and details on adjuvant chemotherapy (CTX) or radiotherapy (RTX). PCV: procarbazine + lomustine + vincristine; CCNU: lomustine; SD: standard deviation.

**Table 3:** Receiver operating characteristics (ROC) analysis.

ROC Analysis	CONV Reading		SM Reading	
	R1	R2	R1	R2
**True positive**	45	43	60	60
**True negative**	40	39	39	40
**False positive**	0	1	1	0
**False negative**	15	17	0	0
**Sensitivity**	73.3%		99.9%	
**Specificity**	98.8%		98.1%	
**Positive predictive value**	98.9%		98.8%	
**Negative predictive value**	71.2%		99.9%	

This table depicts the results of the ROC analysis separately for both readers (R1 and R2) as well as on average for both readers together, considering the whole study cohort consisting of 100 patients. The following definitions were used: true positive = progressive fluid attenuated inversion recovery (FLAIR) signal in gold-standard (GS) reading and conventional (CONV) or subtraction map (SM) reading; true negative = stable FLAIR signal in GS reading and CONV or SM reading; false positive = stable FLAIR signal in GS reading and progressive FLAIR signal in CONV or SM reading; false negative = progressive FLAIR signal in GS reading and stable FLAIR signal in CONV or SM reading.

## Abbreviations

3D	Three-dimensional
CE	Conformité Européenne
CONV	Conventional
CTX	Chemotherapy
FDA	Food and Drug Administration
FLAIR	Fluid attenuated inversion recovery
FU	Follow-up
GS	Gold standard
κ	Cohen’s kappa
MRI	Magnetic resonance imaging
LOBI	Longitudinal Brain Imaging
PACS	Picture Archiving and Communication System
PET	Positron emission tomography
R1	Reader 1
R2	Reader 2
ROC	Receiver operating characteristics
RTX	Radiotherapy
SD	Standard deviation
SM	Subtraction map
WHO	World Health Organization
